# Advancing Smart Home Awareness—A Conceptual Computational Modelling Framework for the Execution of Daily Activities of People with Alzheimer’s Disease

**DOI:** 10.3390/s22010166

**Published:** 2021-12-27

**Authors:** Nikolaos Liappas, José Gabriel Teriús-Padrón, Rebeca Isabel García-Betances, María Fernanda Cabrera-Umpiérrez

**Affiliations:** Life Supporting Technologies (LifeSTech), Superior Technical School of Telecommunication Engineers, Universidad Politécnica de Madrid (UPM), Ciudad Universitaria, 28040 Madrid, Spain; jterius@lst.tfo.upm.es (J.G.T.-P.); rgarcia@lst.tfo.upm.es (R.I.G.-B.); chiqui@lst.tfo.upm.es (M.F.C.-U.)

**Keywords:** memory impairment, dementia, Alzheimer’s disease, smart homes, cognitive architecture, modelling, daily living activities

## Abstract

Utilizing context-aware tools in smart homes (SH) helps to incorporate higher quality interaction paradigms between the house and specific groups of users such as people with Alzheimer’s disease (AD). One method of delivering these interaction paradigms acceptably and efficiently is through context processing the behavior of the residents within the SH. Predicting human behavior and uncertain events is crucial in the prevention of upcoming missteps and confusion when people with AD perform their daily activities. Modelling human behavior and mental states using cognitive architectures produces computational models capable of replicating real use case scenarios. In this way, SHs can reinforce the execution of daily activities effectively once they acquire adequate awareness about the missteps, interruptions, memory problems, and unpredictable events that can arise during the daily life of a person living with cognitive deterioration. This paper presents a conceptual computational framework for the modelling of daily living activities of people with AD and their progression through different stages of AD. Simulations and initial results demonstrate that it is feasible to effectively estimate and predict common errors and behaviors in the execution of daily activities under specific assessment tests.

## 1. Introduction

The elderly population is increasing sharply, and even though most elderly people face the ageing process normally there are specific groups that face different health challenges. The number and the severity of these challenges are increasing, with the most common being chronic health conditions, mental health conditions, physical injuries, cognitive health, malnutrition, social isolation, and cancer, among others [1]. All these challenges stand as obstacles to normal, independent living. For example, impaired cognitive memory affects a person’s ability to think, learn, remember, and perform actions in their everyday life. A considerable number of chronic diseases, such as AD, are increasing in their preponderance due to global population tendencies and will cause numerous problems in the future.

Dementia and AD are two different terms that are utilized broadly and are confused when used in similar contexts [2]. The former refers to a set of symptoms or brain disorders that negatively impact memory, performance in daily activities, social and communication skills, decision making, emotion control, reasoning, etc. All these symptoms affect cognitive functioning. The latter (AD) is one specific such disorder that progresses over time, usually affecting memory and thinking in elderly people over 65 years old. Common symptoms of AD include memory problems, concentration problems, aggression, depression, and confusion, among others [3]. Doctors face difficulties determining whether someone suffers from AD or another type of dementia and currently there are no effective cures. Specific types of dementia respond to some treatments that can ease or stabilize the symptoms, preventing the condition from getting worse. AD is the most common cause of dementia. The risk of developing AD is high in the elderly population and people with cognitive impairment will usually progress to AD [4]. In addition, during normal ageing some elderly people will develop cognitive changes or a decline that will be noticeable in their environment but will not affect their ability to carry out their everyday activities. This is referred to as mild cognitive impairment (MCI) in the literature and can lead to a specific type of dementia or AD or sometimes can revert to normal cognition [5].

In AD, deficits in cognitive function can be found in working memory, task sequencing, attention, speed of processing, orientation, recalling, learning new things, etc. [6,7,8,9]. These deficits affect individuals in a way whereby they require more physical help from family or a caregiver to perform basic activities of daily living (ADLs) or instrumental activities of daily living (IADLs) as the disease progresses. These activities involve most of their everyday tasks and include toileting and bathing, dressing up, feeding, receiving medication, maintaining their finances, or communication tasks.

AD patients wish to be cared for at home, be independent as much as possible, and avoid institutionalization. A report from the United Kingdom detailed that eighty-five percent of people with dementia would prefer to remain at home following their diagnosis [10]. An important segment of these people cannot afford professional care. “Aging in place” as a subdomain of healthcare stresses the need for physical environments capable of enabling the elderly to maintain their full potential [11].

The most common type of caregiver is a family member, and it is typical for people with AD to be cared for at home. Nonetheless, these family members often experience tough burdens and negative physical, psychological, and socio-economical effects [12]. These burdens are obstacles to their normal way of living, but for many socio-economic reasons they need to take care of their loved ones.

SH and emerging technologies have considerable advantages as opposed to caring centers and institutions as they can enhance the independence of the elderly within the early stages or at a moderate stage of AD without the need for professional help. SH technology relies on systems such as telemonitoring, activity recognition, safety mechanisms, enhancement of cognitive performance, behavior analysis, etc. [13]. Consequently, simulating and predicting the behavior of people with AD introduces a great opportunity for developers and researchers to design and develop interventions able to adapt dynamically to the behavior of people with cognitive impairment and to improve activity recognition systems by predicting common mistakes in impaired behavior. Attempts to accurately recognize activities and common routines of people with AD are usually performed by sensory data analysis with different techniques such as machine learning (ML) and recurrent neural networks [14,15]. These activity recognition systems predict behavior at an abstract level and generate outcomes related to the activity or the action someone is performing. However, they are unable to predict human thought and its interruptions when someone has cognitive problems. Combining predictions of human behavior with sensory-generated predictions will lead to advanced prediction systems that can ultimately help smart homes to provide better-adapted interventions and awareness for this specific target user group.

One of the prevailing ways to model human behavior is by utilizing cognitive architectures. They have been used extensively for many years in various research domains such as memory research, artificial intelligence, problem-solving, and decision making, and they all tend to summarize elements and frameworks of cognitive phycology to produce formalized models such as computer programs and generate intelligent behavior in complex environments. Cognitive architectures are classified as symbolic, connectionist, and hybrid [16]. They are all sets of modules that interact with each other and simulate how the brain processes information. The main principle behind cognitive architectures is that they can be utilized to represent task-independent constraints in a programmed way.

State of the art in cognitive architectures include SOAR [17] and the adaptive control of thought-rational (ACT-R) [18]. The first is high-level oriented with lower cognitive level fidelity, making it less suitable for predicting human behavior based on cognitive errors and limitations at the process level. Nonetheless, many other architectures exist but are not so distinguished [19]. EPIC [20] is another example of a cognitive architecture that is suitable for multiple-task performance using well developed perceptual and motor modules. However, it is not goal-oriented, there are no learning capabilities, and it is poorly documented. Turnbull et al. [21] evaluated these three architectures in terms of how suitable and mature they are for use when assessing notification systems. They pointed out that each of them concentrates on different tasks, with ACT-R and SOAR being more suitable for human–computer interaction (HCI) tasks while EPIC is more suitable for simpler tasks and multi-tasking.

Cognitive architectures such as the ACT-R framework provide a toolbox of computational methods that can describe the cognition of humans in a behavior-predicting manner. They achieve this by incorporating general theories of cognition that have been developed over the last century. The main use of ACT-R within the domain of cognitive psychology is to model problem solving, perception, attention, language, learning, memory, etc. Lately, cognitive architectures have been very beneficial in the domain of HCI to assess interfaces under different conditions (e.g., dementia) [22]. García-Betances et al. [23] defined parametric cognitive virtual user models that describe interactions with people with disability-derived functional impairments with information and computer technology (ICT) applications. On the other hand, models have been extensively exploited in the education domain to produce cognitive tutoring systems, such as systems that are able to understand the possible difficulties of students [24]. ACT-R is a cognitive architecture that is based on the ACT-R theory. The latter originates from cognitive psychology experiments and observations. From the outside, the framework appears as a programming language that reflects assumptions about human cognition. These actions are required to enable researchers and developers who are starting to use the framework of ACT-R to:

- learn the coding language and style; this can vary depending on the language they are using; nowadays there are useful ports in different languages, such as Java, Python, Docker instances, etc. The main framework originated from Lisp. However, since each ACT-R model predicts different elements of cognition, it is wise to consider studying similar peer-reviewed models to understand the approach being followed.
- understand specific cognition elements that are essential such as working memory or long memory in humans, memory retrieval in the brain, etc.
- compare with similar models if they exist or to compare and assess with human experiments.

ACT-R operates on different modules and all of them communicate with each other. Each module is responsible for handling a specific type of information. The communication between the modules is achieved only through buffers. The main modules are the visual, the perceptual–motor, the procedural memory (with the pattern matching), and the declarative memory. The procedural module is responsible for accessing the information of the other modules. The cognition layer consists of the production module with the declarative memory module. These modules correspond to the different cognitive functions of the brain.

### Related Work

Recent efforts focus on the decision-making process for smart healthcare environments. Neto et al. [25] designed an offloading IoT-based novel algorithm for face recognition and fall detection. They explored the data provision in a microfog to synchronize the data processing and improve the decision-making process. Goncalves et al. [26] explored the mapping between the physical and emotional state of home-cared-for users and applied this to the elderly population. They considered a participatory design and included the characteristics of the users, including their social, phycological, and therapeutic elements. The solution can identify the behavior of the users and act accordingly by notifying caregivers in the case of abnormal situations. Filho et al. [27] presented an automation decision system based on neural networks that can improve the accuracy of decisions and minimize the energy consumption of intelligent infrastructures by utilizing wireless sensors and actuators.

Attempts to use frameworks and systems utilizing cognitive modelling in the literature exist but are limited. They usually provide coaching systems able to predict normal or erroneous user behavior or they implement context-aware solutions in smart environments to provide better sensing solutions. Amato et al. [28] introduced a personalized coaching system based on cognitive modelling by utilizing ACT-R, which relies on problem solving elements. The main novelty of the coaching system is the fact that the system can handle dangerous situations and behaviors such as when cognitively impaired people perform activities with kitchen objects and manipulate devices such as home appliances (e.g., the oven). Likewise, as a coach it guides patients suffering predominantly from dementia to perform better specific actions. Shaukat et al. [29] mentioned the importance of having and implementing solutions that do not require training data, as they pose ethical, legal, and economic issues, and implemented a solution for people with dementia (PwD) that can provide synthetic data through cognitive modelling. These data are used to evaluate and assess existing assistive solutions through behavior and navigation modelling. Wilson and Turner [30] created artificial agents that simulate generic erroneous behaviors and are focused on anticipated contextual changes in the environment. Yordanova and Kirste [31] argued that existing activity recognition systems rely on probabilistic symbolic models that use only the designer’s intuition and trial and error and that they lack a structured methodology and knowledge on how to implement such performance models. As such, they provide guidelines based on the waterfall model about how to build powerful models with satisfactory results. Recently, Zaedally and Bello [32] summarized the importance of the Internet of Things (IoT) in healthcare and provided an extensive review on existing IoT-based healthcare architecture solutions. They proposed an architecture for a coaching system for daily activities. The application logic of this architecture utilizes ACT-R as the core element that can be used as a layer to highlight dangerous situations or propose behavioral corrections.

Experiments have been carried out in the field of mobile health and active assisted living utilizing cognitive architectures to predict human behavior with computational models. Pirolli et al. [33] developed an mHealth smartphone-based coaching system that was able to support healthy behavior change in diet and stress by using behavioral goals and predicting long term behaviors. Baretta et al. [34] presented a mobile application with a wearable system that targets physical activity promotion by using an adaptive hybrid model that integrates the self-efficacy beliefs and physical activity behavior of the user. Serna et al. [35] simulated the decrease in the execution performance of a kitchen-related activity during the progression of AD and the amount of help required by the caregivers to maintain the execution of this activity. This is the only attempt made in this domain to simulate the cognitive decline across all AD levels. Morita et al. [36] created a reminiscence-based model for the mental care of the elderly. This model uses old photos as memories and generates similar reminiscence behavior. The outcome can be used to motivate the users by guiding their memory recall with photos of healthy user modelling. Furthermore, decision making as a process in the older population has been researched with cognitive architectures and the results demonstrated that the decline in specific cognitive functions contributes to the general decline in strategy execution and decision making [37].

The present study aims to deliver a set of models packaged as an easily extensible framework, which serves as a context-aware tool and provides better accessibility support for designers and developers when they design ICT healthcare solutions for people with AD. Our interest domain is the smart home area, and this framework will serve as a base for predicting the behavior of people with various levels of AD when executing daily activities.

The novelty of the framework and its contribution are of paramount importance and can be summarized as follows:

- There have been no similar frameworks in the literature in recent years dealing specifically with daily activity modelling and involving state of mind analysis and modelling. Serna et al. [35] (2007) most recently produced a simulation of a decrease in cognitive functioning across the AD spectrum when executing one kitchen task.
- The modelling of cognitive deterioration in the literature does not include phycological elements as we analyze later, except the simulation of Serna et al. [35]. To our knowledge, there are no recent attempts to simulate how much support is needed by incorporating phycological assessments. This is necessary to provide an alternative view to ICT stakeholders involved in the process of designing an intervention.
- There is no existing framework that combines several daily activities. We introduce three daily activities in this study and we plan to incorporate more in the upcoming version.
- It is imperative to keep producing and maintaining computational models as opposed to modern techniques that require a large amount of training data. Neural networks would require huge fMRI datasets to perform the same predictions, which would be almost impossible to gather.
- This framework can be combined with activity recognition systems to conduct a futuristic detailed state of mind analysis when performing daily activities. We believe in the paradigms of “Personalized Medicine” and “Aging in Place” as future-proof senior living arrangements and this framework is a milestone towards better dynamic adapter interventions.
- Considering future plans, more insights will be discovered when new assessment tests are incorporated into these models. In addition, more dementia types should be added to model different situations.

The following sections are organized as follows: Section 2 presents the value cognitive models bring to the awareness of SHs, and introduces a generic description of the framework with the functional tests; Section 3 delivers the methodology and the implementation details; Section 4 gives an overview, runs through the details, and looks at how our method is different from previous models; Section 5 depicts our initial results and validation; and Section 6 delivers our conclusions and discusses our future plans.

## 2. Advancing the Awareness of SHs with Cognitive Models

SHs can provide assistance in the autonomous completion of various tasks by either detecting abnormal behavior and informing a caregiver or by telemonitoring and aiding people from a distance using telemedicine techniques. Predicting the behavior of people with cognitive deficits caused by AD is an important milestone towards the homogenization of emerging technologies, smart homes, and cognitive assistance, with the end target being to provide automated, dynamic, personalized interventions. The researchers and the developers must be aware of the status of the cognition of people with cognitive problems in order to perform quantitative predictions regarding latencies and accuracies in their activities in real life. The end goal is to provide a framework for researchers and developers to exploit when designing superior adapted interventions. The more personalized, adapted, and dynamic the intervention is for the final user, the higher the approval rate will be—meaning that the intervention can be adopted by the user adequately. The proposed conceptual framework acts as a context-aware tool that provides valuable information to the intervention designers and researchers.

This paper aims to demonstrate a computational parametric framework that is able to simulate the cognition sequence and errors during the execution of an ADL or an IADL. Hence, the framework currently consists of three daily activities’ models and the common input parameters that the user can modify and run in each model separately. This framework serves as a base that will be further expanded to create more complicated environments of daily activities in the context of SHs. The models provide the necessary information needed by smart home developers and researchers to adapt consistently and enhance or provide new interaction paradigms that can be understood by the final users. Eventually, the SH will act dynamically and automatically and will control the devices and home appliances on behalf of the users. Furthermore, the framework can be used to enhance standard artificial intelligence (AI) machine learning activity recognition algorithms and produce a top-level advanced context-aware framework for the house for this specific group of users.

The implemented models as of now are tea preparation activity, washing hands activity, and dressing activity. Research has been undertaken showing that different parts of the brain control different activities and perform different actions [38,39]. Hence, depending on the level of cognition deterioration, each user will have slightly different symptoms. However, memory problems are the predominant issue that occurs at most AD levels. Hence, we proceed mainly with the memory and functional behavior deficits of AD (e.g., repeating actions) as they are the most common. We chose to model these three important activities as users find it difficult to cope with and caregivers also find it challenging to assist them with these activities if they do not have the proper training required and adequate time for them. The washing hand activity was chosen as it is a typical routine for someone during the day and belongs to the basic daily care plan of caregivers. The dressing activity was chosen as it is affected more at the later stages of AD and is an action that people with AD are ashamed to admit that they require assistance with. The tea preparation activity was chosen as a base activity as it belongs to the cooking and kitchen-related activities category and there are suitable occupational therapy tests available in order to help us expose common errors.

An important element that is necessary to comprehend is the way occupational therapists assess functional performance, disability and its severity, and the amount of help needed by people when they perform their daily activities. The current way to do this is by utilizing functional tests designed and scientifically approved to generate outcomes based on the performance of the participants. Some of the available tests are the E-ADL test [40], the Arnadottir OT-ADL neurobehavioral evaluation (A-ONE) test [41], the executive function performance test (EFPT) [42], and the kitchen task assessment test (KTA) [43]. Accordingly, our models incorporate psychological observations from EFPT and KTA that can translate all problems into an abstract level of different categorized problems. Finally, yet importantly, it is the base of our evaluation technique since we are comparing the mean and standard deviation (SD) results to the KTA [43] and the ACT-R model developed by Serna et al. [35] in a similar pattern.

Parametrizing the models with the ACT-R input values allows us to simulate all the levels of AD and produce the simulated behavior across all the stages and receive valuable information regarding the sequence execution of daily activities. The outcome is a close mind step analysis of human thought and production-style sequences as executed by the models. The output execution of each model provides common errors found in different categories as exposed by the functional test of EFPT and KTA. The models can be executed by as many people as are given as inputs.

### Incorporating Phycological Properties in the Models

The executive functional tests assess different skills such as working memory, organization skills, attention, concept formation, inhibitory control, etc. Different tests exist in the neuropsychology domain and each one aims to evaluate a group of skills [44]. They are performed in real settings, usually require 30–60 min to administer, and are widely used to discover functional problems in different populations such as children with learning difficulties, elderly people with cognitive problems, people who were treated for cancer and developed cognitive problems, or patients who suffered a stroke.

As mentioned before, the models of this framework incorporate psychological properties found in common assessment tests, namely EFPT [45] and KTA [43]. The EFPT (executive function performance test) is designed to evaluate executive functioning in these four tasks: cooking, bill payment, telephone usage, and medication management. EFPT is a performance-based standardized assessment for cognitive function using instrumental activities of daily living. It uses a top-down approach in real world settings and can identify the impaired cognitive functions, the capacity of the person to function independently, and the amount of help needed to complete a task. This test originated from occupational therapists (OTs) as a means of observing and monitoring performance in daily activities. It was initially used in stroke patients [46]. The kitchen task assessment (KTA) is a similar test to the EFPT. However, it is more oriented towards people with senile dementia of the Alzheimer’s type (SDAT) and is used to evaluate their cognitive functions [47]. The test asks the participants to cook a package of pudding and it measures the changes in performance. This is a standardized test for patients with AD to assess their cognitive abilities when they perform their daily activities. Likewise, it is used to record the cognitive changes during the progression of AD. Both tests operate similarly and mainly examine these cognitive functions: initiation, organization, performance of all steps, sequencing, judgment and safety, and completion. Each of these categories receives a score depending on the amount of help required to complete a step of a specific task. Table 1 depicts the scoring according to the official KTA test and describes how each category is assessed [43].

As such, the maximum score this functional test can obtain is 18 points (summing up the score of each one of the six cognitive functions). The higher the score, the more cognitively impaired/incapable the person is and hence they require more help. For instance, a person with mild AD would score an average of 4–5 points on one activity, as can be seen later in our validation in Section 5.2. These tests provide realistic scenarios on the amount of help someone requires. The SH solutions should incorporate this psychometric data to better contextualize the environment and provide better dynamic interventions.

## 3. Methods and Implementation

The models simulate activities employing different sequences of steps and execution stages to discover common cognitive and behavioral errors that people with AD make. It is essential to model the most important cognitive and behavioral errors that these people perform. A top-down approach is applied in order to break down the most essential elements required for this framework. The elements identified are the exact cognitive processes involved when performing activities, the view of the problems from a caregiver perspective, and the occupational therapy tests focusing on the cognitive aspects only that do not require further motor or perceptual skills to assess. Since the brain operates in different domains such as smell, motor, etc., when we describe the cognitive processing of the brain, we focus mostly on the information flow rather than the motor skills and how the brain uses the information to operate effectively in the world by taking the information as an input and processing it in a continuous way that enables humans to interact with the world [48]. For example, an activity of receiving medication involves steps such as looking for a specific medicine, choosing one from among similar medicines, preparing some water, taking the medicine, and finishing the task. These operations are cognitive processing actions, and the models work similarly. We incorporate in our model the EFPT test coupled with the KTA. Both tests assess the task completion problems that someone can face during the execution of daily activities. We utilized KTA as a validated information source that presents us with the most common mistakes that people with AD make when they cook. Every problem that occurs during the execution of an activity belongs to one or more of these judgment components (e.g., organization problems), as explained previously.

**Figure 1:**
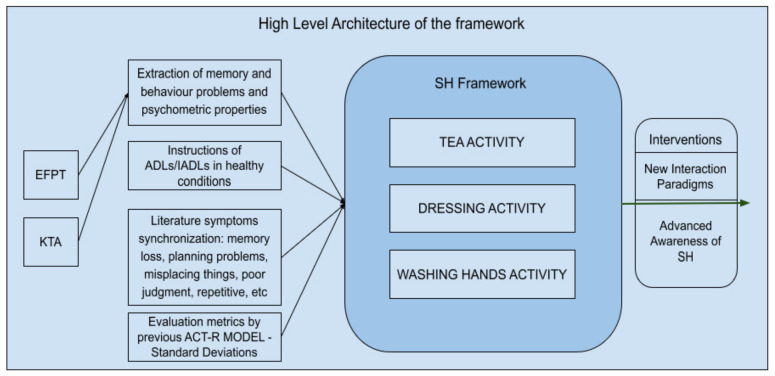
High-level architecture of the framework and the methodology overview.

The methodology followed was based on these steps: (1) model a healthy person performing the ADL as a sequence of steps with different stages utilizing the goal-oriented design paradigm [49]. The steps utilized in the activities are normal behavior steps someone has to follow to perform an activity such as dressing. For example, in the case of dressing activity: one has to find an upper dressing item in the environment, apply it, finish this action, complete this stage, and move on to the next action such as finding a low dressing item (e.g., trousers). These steps are applicable and typical in occupational therapy or physical therapy sessions (e.g., for stroke or brain damage or physical damage) and in rehabilitation techniques [50,51]; (2) inject the memory errors (forgetfulness and similarity of items, according to the most common errors made in KTA, and according to the EFPT error categories); (3) incorporate the memory behavior errors (based on the common errors people with generic cognitive impairments (MCI or dementia or AD) face, such as repetitive tasks, not able to start/finish tasks, and confusion of the sequence of tasks of an activity); (4) model the help provided by the caregiver to the user according to the EFPT/KTA; (5) parse the results by incorporating the occupational therapy assessment category problems into the models; (6) analyze the results according to the type of problem that occurred; (7) propose interventions in the scope of SHs according to the misbehavior and the severity of the AD. The structure of the steps of each model favors a modelling method with a goal-oriented design paradigm where each step is modelled as a goal (a production in the ACT-R terms) and this goal can be suspended, executed, repeated, or confused with other steps. Figure 1 depicts the high-level framework architecture for a better understanding.

### 3.1. Describing Intelligence through Cognitive Modelling

The general goal of AI is to create computers able to do “human things” or to explain how intelligence works and re-produce it in the computer or within simulated environments. Since the early stages of computer science and finite-state machines, AI has continually evolved towards human intelligence. In the cognitive theories, the models try to reproduce this human intelligence in simulated environments and exploit it in favor of different domains of science such as the health domain or the biomedical domain. Principally, a cognitive model is a mathematical interpretation of a set of principles that originate from a theory of cognition. This interpretation enables us to make qualitative and quantitative measurements that are comparable to measurements from human participants through experiments. Memory disorders interfere with many aspects of a person’s life such as recalling, learning new things, triggering apathy, paying attention, recalling, etc. The framework of ACT-R includes aspects of both symbolic and sub-symbolic theory. These aspects are essential to represent the cognition of humans on an intellectual continuum. In addition, sometimes they refer to as “connectionist systems” and they include the metaphors of the neurons, meaning, they are a collection of a small perceptron that operate in parallel in the brain to recognize the input [52]. Nowadays, the sub-symbolic systems are used in modern AI systems to achieve more robustness against noise and for better performance through deep learning, ML, and neural networks. Symbolic systems may refer to explicit symbolic programming, rules, ontologies, plans, etc., whereas sub-symbolic systems refer to systems such as Bayesian learning, deep learning, big data, neural nets, etc.

The sub-symbolic system with the production system of ACT-R enables us to go through alternative directions when developing predictive realistic behavioral models. A misconception arises here, and someone might argue that nowadays the paradigm of machine learning can produce more accurate results regarding the modelling of behavior. Nonetheless, we are modelling people with AD focusing on their memory and thus it is impossible to gather such big datasets for supervised learning activity simulations and predictions. ML and deep learning techniques can successfully model the generic tasks with sensors (e.g., detect sleep activity and patterns in SH) or they can predict early symptoms such as changes in the brain analyzing MRI images with great success, but they are unable to reproduce the execution of the steps and the cognition sequence.

The framework models and operates through the whole spectrum of AD, focusing on the memory aspects and the psychometric properties as exposed by the functional tests. The starting point is the early cognitive impairments that may arise as a pre-AD stage (such as MCI) and the endpoint is severe AD. This is the progressive spectrum of the disease achieved by modelling the working memory of users when they perform daily activities [53].

The mechanism to model short and working memory in ACT-R is realized by the concept of activation [35,54]. The memory module of ACT-R, namely the “declarative memory”, holds basic information such as facts known to the user. Combining the declarative memory of ACT-R with the concept of activation gives the working memory concept. Declarative memory can hold a lot of information but only the information that has an activation value greater than the threshold can be retrieved and used by the subject (activation concept). The information is represented by the concept of chunks. A chunk is the smallest piece of information that the memory module of ACT-R can contain and remember. Using this mechanism of information representation, we can transform the memory into a semantic network of information modelled and accessed only in certain circumstances depending on the activation values. For impaired cognition, this network of chunks is usually broken, difficult to retrieve, or confused by other similar chunks of information. Furthermore, in cognitively impaired users we ought to model incorrect and unexpected behavior. The appropriate mechanism to implement this in ACT-R is by creating conflict situations for the actions that the users perform. These two mechanisms play a major role in implementing our models, which are described later in more detail.

The conceptual framework currently contains three daily activities and can be easily extended. To be more precise, we modelled one IADL activity (tea preparation) and two ADL activities (washing hands and dressing). As mentioned before, the chosen base activity modelled was the “tea preparation” and this activity benefits from an adequate number of retrievals of the memory module, and we are interested in validating the results with real user data at a later stage. In addition, the tea preparation activity being a kitchen-related activity helped us to better identify the problems by exploiting previous research [35,43] and thus maintain a balance between memory errors and behavior conflicts. The sequence of actions and all the stages of tea preparation are based on the recipes’ instructions that can be found on commercial packages over the market. Future design considerations as part of our methodology are to assess possible amendments to the cognitive parameters when switching from ADL to IADL and possible adjustments to the framework output to provide different results within different dementia types (this might require the modelling of the surrounding environment of the patient as well as the use of perceptual motor modules).

### 3.2. Modelling Cognitive Errors—Organizational and Behavior Errors

The ACT-R framework is suitable for modelling cognition, the flow of information, and errors using either its sub-symbolic system with the production system or the concept of activation. The errors related to cognitive deterioration are differentiated roughly into three categories according to the nature of the ACT-R and its modules. These categories are omission errors, commission errors, and behavior errors. The omission and commission errors correspond to errors related to the semantic memory exclusively and are hence organizational errors (forgetfulness and confusion by similarity), while behavioral errors refer to cognitive errors that affect behavior. For instance, to confuse the sequence of the steps, confuse the different stages of the activity, not execute specific steps, or not start the activity at all is a behavioral error. Modelling these three categories of errors is enough to provide a broad picture of how the cognition flow works and how and when the user is going to fail when performing specific tasks. This provides adequate information and awareness to the SH for the designers to provide more patient-centric interventions and combine the new interaction paradigms more successfully. As a consequence, we chose to model using only the memory module of ACT-R and avoid using other modules such as the perceptual-motor module because the hypothesis is that cognition and memory are responsible for the majority of the errors in persons with AD and is present in all stages, whilst at the same time most cognitive rehabilitation techniques target memory deficits. Moreover, the end framework must be simple, easy to understand, and extensible for other researchers to use and further develop.

**Figure 2:**
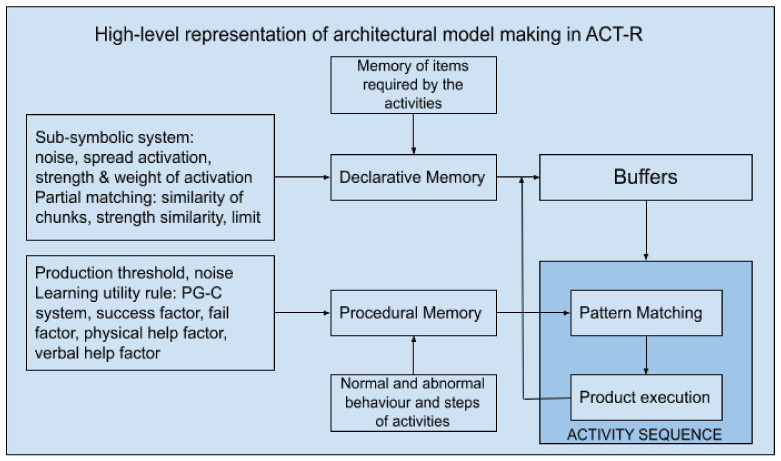
High-level representation of architectural model making in ACT-R.

Figure 2 depicts the high-level architectural model-making process we followed and all the mechanisms involved in the models as well as the parameters that the framework requires to work. The users are free to introduce their own parameters as inputs into the models and observe the difference in the output results. However, the default parameters are already pre-defined in the framework as a result of evaluation-matching with the scores of previous attempts at the KTA and a previous ACT-R model. An important element to note is that through the simple graphical interface we provide this framework can be used without programming knowledge, which perfectly satisfies the designers’ needs or those of other stakeholders involved in the design process of these interventions.

As mentioned previously, this conceptual framework models the errors of people with AD within different daily activities and across different levels of AD. Additionally, it models the help provided by the caregiver according to the functional tests. The procedure and the mechanism to model the omission and commission errors is the same, and we accordingly modify the parameters of ACT-R to achieve greater decline and simulate different levels of cognition deterioration (MCI, mild AD, moderate AD, severe AD). During the execution of the daily activity, the user forgets specific elements caused by the decline in their working memory. The category of omission errors corresponds to the errors by the user when he forgets specific items necessary to complete a task. For example, in the model of the tea preparation, the user will forget some items (e.g., forgets where the tea bag is located) that are needed to complete all the steps. This is an important category of forgetfulness error as without the necessary elements, someone cannot execute any given activity with success. The second type of error is commission errors and corresponds to the errors done when the user confuses two similar items (e.g., mug and kettle) or confuses the locations of different items (this is not represented in the models; however, the similarity of two objects is used and has the same effect). These two categories are very important when modelling cognition in humans and in ACT-R both are modelled by the concept of the calculation of the activation value of chunks in the declarative memory. Every chunk has an activation value. When this value is greater than the predefined modelled threshold then the chunk is retrieved by the memory and the user “remembers” it. Each time the user accesses one chunk of the memory, the activation value gets higher and in the next memory request it will be easier for this specific chunk to be retrieved rather than be confused/forgotten. Manipulating the concept of activation in ACT-R is the way to model omission and commission errors. More specifically, a chunk (*i*), and the given elements (*j*) that are part of the goal/focus chunk, has a total activation value A, which is calculated with the following equation provided by the modules of ACT-R:

(1) $$A_{i}=B_{i}+\overset{n}{\underset{J=1}{\sum }}\frac{W}{n}S_{ji}+\underset{k}{\sum }P_{k}M_{ki}+\varepsilon$$

Each of the four elements in the equation is a modelling mechanism in the world of ACT-R modelling. Every developer and researcher can exploit them and parametrize them as they wish to produce their models as close as possible to the real use case scenarios and environments. This equation is used to create the semantic network of information and chunks with their activation values. The following points describe this equation and its components in more detail (1):

- The first term *B* in Equation (1) describes the following: each element in the declarative memory has a base level activation value B, which is the activation level that can be retrieved and in ACT-R and simulates the process of forgetting and remembering. Every time the chunk is harvested, the total activation attains a higher value. The base-level activation *B* can decay the memory over time, which we are not interested in in the case of AD since their memory is already in decline. In general, the base level activation reflects the usefulness of the chunk in the past and its relevance to the exact context. We do not modify this parameter in our model and we keep it default and stable.
- The second term in Equation (1) is the summation equation of the source spreading activation concept of ACT-R [55]. We make use of this summation parameter as it introduces errors into the memory regarding the association between chunks. This formula is used to increase the activation value of the chunks that are similar to the existing chunks already in the buffers (e.g., if the current goal has a chunk *A* + *B* and the memory module requests chunk *B* then *B* gets a higher activation value and it is more possible to receive it). The *S* term defines the associative strength equation of activation for the buffers and the *W* term defines the attentional weight equation to adjust for how many slots to spread the buffer from. W is usually set to 1, so the attentional weight is 1/*n*, where *n* is the number of sources/terms. *S* is usually 2 and represents the number of facts associated with the chunk. This is the concept that creates the semantic network of chunks in memory, and similar chunks might be confused and forgotten when noise is introduced. In our models, we keep the *S* with a stable value of 2 and we modify the *W* parameter by reducing it at each progressive stage of AD to achieve higher cognitive decline regarding the association of chunks and goals; a lower value of *W* translates to problems with chunk association and by extension information retrieval.
- The third summation in Equation (1) is the partial matching concept of ACT-R [56]. With this mechanism, we introduce similarities between chunks in memory. For instance, the kettle and the mug are very similar in the model of the tea preparation. The matching scale *P* defines the strength of similarity, and the *M* defines the similarity between two chunks. We use both parameters to define which chunks should look similar and their strengths.
- The last term in the equation is also very important as it introduces the noise required for the memory to create parametrized decline—to simulate different levels of AD.

The other mechanism being utilized, as can been seen in Figure 2, is the production system with the sub-symbolic processes offered by ACT-R. The behavior errors are exposed through occupational therapy tests and are common errors that confuse the behavior of people with cognitive problems. ACT-R uses a production system paradigm [57], which can be parametrized to have many productions available to run at the same time.

This creates conflicts in the sequence of tasks and produces behavior errors. Each time only one production runs (step), but we configure the models to have more than one run at the same time; the model must choose which one to run every time. Each production has a utility value, which is calculated by the probability of successes and failures with additional noise; the higher the utility value, the higher chances the production will fire. We exploit this mechanism to produce conflict situations with the users. We predefine the utility values for specific productions to run. Having a parameter pre-defined before the model run, we define the values of how often we desire the subject to confuse the productions. The utility value can change on the fly while the model is executing depending on the production that is executed. Finally, this mechanism enables our framework to cover the different stages of cognitive deterioration within the different AD levels, parametrizing the utility values of the production system. More specifically, we use the classic “PG-C” system of ACT-R; each utility value is responsible for determining which productions are selected when a conflict appears. The utility value of a production *i* is calculated by the following equation:

(2) $$U_{i}=P_{i}G-C_{i}+\varepsilon$$

where *P* defines the expected probability of a successful firing; *G* is the value of the goal (in ACT-R is in time); *C* is the cost in time; and *ε* is the transitory noise of the production. In our models, we parametrize the utility value *U* to achieve a probabilistic firing of the productions leading to misbehavior. In addition, we add the transitory noise by default in all productions—as the level of cognitive decline progresses, the noise increases.

### 3.3. Modelling the Help Required by a Caregiver

The functional tests require the help of the caregiver to estimate how much help is required and at which stage when the user is performing the activity. In principle, the functional test is based on the level of help needed by the user. There are two kinds of assistance in these functional tests: verbal and physical help. The help required is modelled in a similar way to the errors.

We have two main categories of errors—memory errors (organization errors) and conflict situation errors (all behavior errors). Firstly, help with memory errors is modelled with chunk activation: when a chunk cannot be retrieved because of a failure (forget or confusion with similar items), a new production (“boost activation”) is called and it once again adds the chunk to the declarative memory and makes a new request for the memory. In this way, the activation value of that specific chunk increases and it is easier to retrieve next time without problems. Depending on how many times this production is run (boost activation), we determine whether the help needed is verbal or physical. For example, if the production is required only one time, we interpret it as verbal help, whereas if it is required more than two times it is physical help as the chunk is very difficult retrieve. On the other hand, the behavioral errors are based on the utility values that are given as parameters before the model’s initiation. Each production that fails to run (a conflict), creates new productions that serve as verbal and physical help productions. Both have the same utility values predefined so, for instance, let us assume that the utility value for a production to run is 40 for success and 60 for failure. This production may fail and require help. The new verbal help production will run with the value of 40 on success and 60 on fail. However, we have embedded an extra factor (extra value) that will be added to the verbal help production. This extra factor is to be defined by the user; an increase of 20–30 in the utility value leads to the desired results in the verbal help, depending on the level of the AD. Physical help is modelled similarly by using the production utility values. Table 2 describes two representative steps as an example in the tea preparation activity. The first one is a memory retrieval step where the subject requests an item from his memory, while the second one is a behavior step where the subject simply tries to execute this step and move on to the next one. The steps are production goals that must be executed, and the possible outcomes are given together with the approach with which the help is injected. The last column describes the help interpretation as verbal, physical, or incapable, facilitating the score generation.

## 4. Overview of the Framework

Contextualizing the SH requires an excessive amount of data and specific scientific knowledge to understand and “feel” its users in different situations and moments of their life. Common methods to contextualize the environments include developing activity recognition systems or behavior prediction models. This manuscript presents a scalable conceptual framework targeted towards researchers, professionals, and developers that exploits the common problems of people dealing with AD and memory problems. Currently, the framework holds three activity models that are able to simulate unexpected behavior and errors caused by a cognitive decline when executing them.

Our interest lies in the simulation of the whole spectrum of AD and extends to other dementia types at a later stage; hence, all levels of cognitive deterioration are included according to the Washington University Global Clinical Dementia Rating (CDR) Scale, which is used as a standardized multicenter reference value. We refer to the levels as: MCI or questionable AD (CDR-0.5), Mild AD (CDR-1), Moderate AD (CDR-2), and Severe AD (CDR-3) [58]. The framework is parametric and requires the input of parameters related to declarative memory and procedural memory. The mandatory inputs are the activity, the AD level, and the number of people in the simulation. Using these inputs, the framework produces the suitable behavior as explained in detail previously. No prior programming knowledge is required to use the framework.

**Figure 3:**
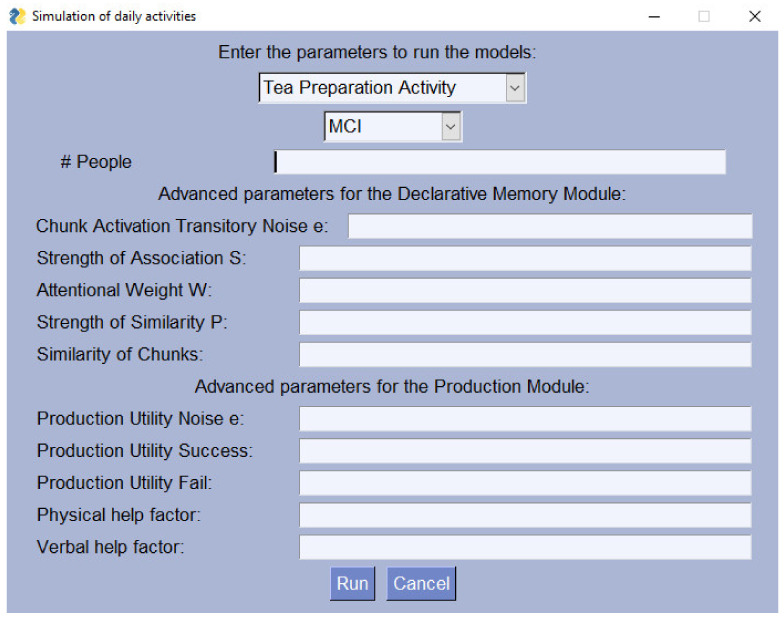
Main graphical interface of the conceptual framework. The required inputs are the activity, the level of AD, and the number of people to run the experiment.

The initial screen of the framework can be seen in Figure 3 as the user is presented with a dropdown menu of the three activities included and the parameters he can introduce. The suggested value for the number of runs is one person or run for 100 people to demonstrate the mean scores. Although the input number can be any given number *n*, we recommend these two options to get different outputs.

**Figure 4:**
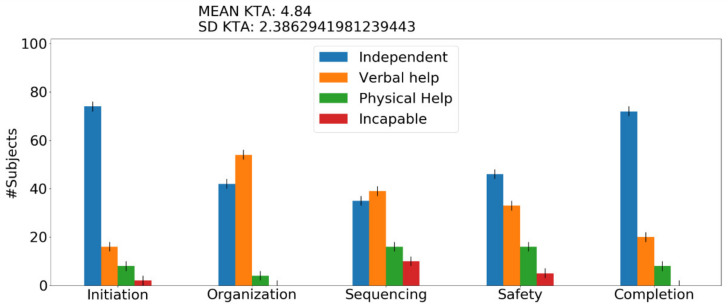
Main graphical output of the conceptual framework. The blue bars represent the number of subjects who completed a category of steps independently. The orange and green bars represent the number of subjects who requested help (verbal or physical) in a category of steps. The red bars indicate the number of subjects who are incapable of performing theses steps under a specific category. This activity corresponds to the tea preparation activity with mild AD.

Running the experiment for one subject provides the score of the assessment test KTA and additional memory problems the user encounters. These additional memory problems refer to the number of times he forgot something in the activity, the number of times he confused similar things, and the number of times he confused an item with a different one. These results are presented in the terminal as a text output, whereas when the experiment runs for more than 1 subjects (e.g., 100 or 1000), a figure is generated that presents the results in the form of assessment categories, as can be seen in Figure 4. Last but not least, a big obstacle observed with ACT-R programming was the unfamiliarity and unwillingness of the researchers to learn and deal with Lisp. The framework was implemented through the Python port of ACT-R to be easily accessible to all operating systems, understandable, and scalable to other researchers since it is a common language that researchers and designers utilize these days. A command-line execution option is also provided.

### Differentiation from Previous Models

The framework currently works with three different activities, simulating abnormal behavior and exposing errors as a form of memory error and EFPT-categorized problems. As stated before, there is a similar cooking model that was made by Serna et al. [35] and the main objective was to simulate the decrease in the cognitive performance across the AD spectrum and the support needed. This admirable work of Serna is a unique attempt in this field of modelling daily activities with the psychometric properties of people with AD, and they developed one cooking activity, which serves as a foundation for us. We go one step further and provide a framework to exploit possible behavioral problems in many daily activities and the help required, ultimately generating dynamic adaptive interventions, in an easy way.

The main differences are listed below. We provide an easy-to-use framework with a simple UI so that no prior programming/terminal knowledge is required to run the software. The designers will be able to run it easily and observe the results. In addition, it automatically generates a graph with categorized errors, which is intuitive. Furthermore, we ultimately plan to incorporate almost all the important daily activities into this framework. From a modelling perspective, we use a slightly different approach to model some situations in the production and memory modules, as described in the previous section. Another element is the assessment test. We chose to go with the EFPT test for problem discovery as it is more generic and can cover more activities in the future. However, we also generated a KTA score as a means of preliminary evaluation as will be described later. Lastly, as a next step, the main outcome of the framework will be a rendered taxonomy of possible interventions and interaction paradigms in a graphical intuitive way targeted for researchers and designers. Future plans are presented in the last section.

## 5. Results

**Figure 5:**
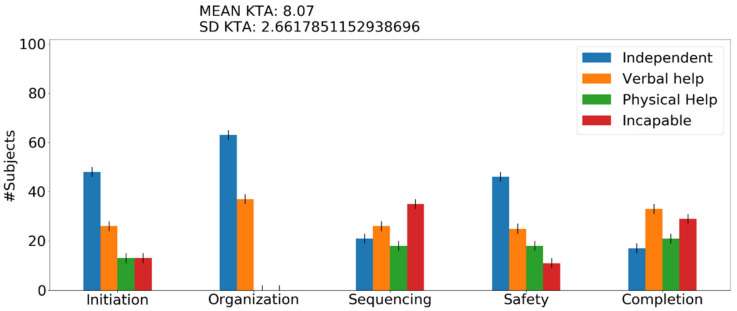
Main graphical output of the conceptual framework. The blue bars represent the number of subjects who completed a category of steps independently. The orange and green bars represent the number of subjects who requested help (verbal or physical) in a category of steps. The red bars indicate the number of subjects who are incapable of performing theses steps under a specific category. This activity corresponds to the washing hands activity with moderate AD.

We demonstrate initial results using the framework to show the memory problems in the daily activities of people with AD. As explained before, the utilization of functional tests provides us with the psychometric properties we aim to exploit. This enables us to connect to the real world and provide better contextualization for future smart homes that can support people with AD. The problems in the daily activities are categorized and presented as follows:

Initiation—problems when the subject cannot initiate the task. Sequencing—problems when the subject confuses the sequence of the stages. Judgment and safety—behavior problems when the subject is in danger (e.g., forgets to turn off a cooking device). Organization—all the omission and commission errors. Completion—when the subject performs one step over and over. In Figure 4 and Figure 5, we demonstrate the figure output of the framework after a model run for 100 people with the activity of tea preparation with mild AD level and after a model run for 100 people with the activity of washing hands with moderate AD level. For each graph output, the KTA score mean and standard deviation (SD) is generated and is used to provide us with comparison information. The graph depicts the number of users *n* out of 100 that required at least some help (e.g., in Figure 4, 40 users required verbal help with their organization problems while 60 users remained independent; this translates to the fact that 40 users needed verbal help at least once—this is the minimum number). When the SD of the KTA score is greater than 2, we display an error bar. We are interested in maintaining the results as close as possible to the mean of the KTA; hence, we add a small error by two users since the minimum error is already depicted in this diagram and we are counting persons.

### 5.1. Smart Homes, Interaction Paradigms and Design Considerations

Technological advances in the domain of SH enable users with AD to leverage interventions that better suit their needs and desires. These interventions will better fulfil the needs of these people, especially the desire to avoid institutionalization, and will reduce the burden on caregivers. In this section, we provide possible interaction paradigms and design recommendations that arise as a result of the problems generated by our framework, as demonstrated previously. Firstly, regarding robotic strategies, they appear to be a well-suited solution to perform a wide range of tasks, such as cognitive training and motivation tasks, and for performing more advanced tasks like assistance with daily activities such as providing medication at set times. For example, an assistive programable humanoid robot can assist in the activity of washing hands by presenting steps and actions required of the user. An example of an interaction paradigm would be a house that can sense when the user is about to initiate the washing hands activity. At that moment, the robot would follow him to the bathroom and assist with steps and items required to successfully perform the daily activity. Likewise, robots have the capability to work as a monitor mechanism and a caregiver will be able to monitor the user at any time if necessary. Furthermore, home automation and digital home assistants nowadays provide an intuitive way to interact with the house and control different appliances, as well as providing reminders, calendars, alarms, and other information critical for people who deal with memory problems.

The conceptual framework presented is an example of the basic awareness the house needs to have to interact better with people who have specific needs, such as memory problems caused by AD. Researchers, designers, and developers often find it challenging to understand these needs and thus their interventions are not well adapted to the needs of this group of users. This framework provides an alternative view of the memory problems someone is facing. Therefore, having run a moderate dementia daily activity simulation, a researcher/developer will keep in mind that safety problems are more important at this stage rather than mild cognitive impairment; thus, the implementation of emerging mechanisms is needed. Additionally, verbal and visual prompts all over the house are the main priority in order to tackle organizational errors and assist the user with daily activities. Moreover, house awareness tips arise as a result of this framework’s outputs. As an example, the developers will be aware of which AD levels present people with more organizational problems and thus their interventions will be more adapted to the needs of the user. Lastly, design considerations also arise as a result of the simulation of these models. For instance, in the case of severe cognitive decline, users face initiation problems with one activity most of the time. This leads to anxiety, depression, and emotional challenges that the developer can tackle when he is designing his interventions. The main contribution of this scalable conceptual framework is the provision of information across different daily activities that enables future smart homes to genuinely serve the user, improve his quality of life, and avoid the burden of caregiving or institutionalization.

### 5.2. Validation with Previous Models

The framework presents quantitative categorized outcomes related to cognitive problems caused by AD at different levels. At this stage of the framework, the quantitative outcomes are the only results depicted in the graphs with the terminal providing some more memory-specific (omission and commission) errors. As such, the experimental validation requires us to run the EFPT or the KTA test with real users and compare the results to those of our model. These functional tests act as a bridge to validate the effectiveness of the framework. Hence, an experiment with 10 users with mild AD would need to be conducted and their results would need to be compared to those of a 10-user simulation. The correlation of the scores leads to the validation of the models at an adequate preliminary level. Nonetheless, due to various difficulties caused by the pandemic, it is difficult to perform experiments with this particular group of people at present. We proceeded to compare our results with the unique previous attempt made by Serna et al. in his model of a cooking activity with the KTA validated test with 106 real users. The mean scores demonstrate the strong correlation between our framework and the model by Serna et al. (r = 0.99). Table 3 presents the mean values and the standard deviation of KTA scores at all levels of AD, obtained by KTA, Serna et al.’s model, and our model (using the tea preparation activity). It can be seen that the models of our framework provide good results that can serve as pre-validation. In fact, the mean results of KTA have been used as the groundwork to start forming models and injecting errors, as explained earlier. We identify the limitations of this validation as the KTA does not provide a distribution of errors between stages or among different criteria. Nonetheless, the aim of this framework is to give an overall image to researchers, stakeholders, and designers of interventions of how AD patients face cognitive problems in many of their everyday activities and to provide a better contextualization of a future smart house that can provide advanced interaction paradigms.

## 6. Conclusions

We presented a conceptual computation framework that simulates common cognitive errors committed by people with AD when performing a daily activity. The initial results demonstrate that specific errors are committed more frequently, especially in the organization category, which is responsible for all the memory retrieval requests in the sequencing stages of the tasks. The purpose is to deploy an awareness framework targeted towards developers of SHs to implement adapted dynamic interventions by perceiving the most common cognitive errors of people with AD. Although the framework has not been fully validated with real users yet, the pre-evaluation results and score matching with the mean and SD of KTA, which was validated with 106 real users with senile dementia, provide us with confidence to develop this framework further and contribute to future dynamic interventions of technologically advanced smart homes. Future plans include:

- Validation with users in our smart home.
- The inclusion of more activities.
- A rendered taxonomy of possible interventions and paradigms as the standard output of the framework.
- The incorporation of extra occupational therapy tests or performance/functional tests, such as the E-ADL test [59] and the cognitive performance test (CPT) [60], in the modelling process, which will give more insights into the help required.
- The combination of activity recognition and coaching systems for a complete dynamic provision of well-suited interventions.

This manuscript delivered a set of models combined as a framework with a simple UI to create a useful simulation of the daily activities of people with cognitive deterioration caused by AD. The objective of the conceptual framework was to help all stakeholders involved in the process of designing interventions to better understand the memory problems that arise during the completion of daily activities and to contextualize the behavior of people with cognitive problems. At this early stage, the framework contains three imperative activities, which are tea preparation, washing hands, and dressing. The ultimate target is to provide a framework that can reinforce the execution of daily activities with assistive technologies by having adequate awareness of the missteps, interruptions, and unpredictable events involved in the execution of various activities of people with cognitive problems caused by AD.

## Figures and Tables

**Table 1:** The KTA functional test with the scoring elements.

Component	Independent	Required Verbal Clues	Required Physical Assistance	Not Capable
Initiation: Can the person begin the task?	0	1	2	3
Organization: Can the person gather all necessary items to perform the activity?	0	1	2	3
Performs all steps: Can the person perform all the steps necessary to complete the task?	0	1	2	3
Sequencing: Can the person perform the actions in the correct sequence?	0	1	2	3
Judgment and Safety: Does the person perform the tasks safely?	0	1	2	3
Completion: Does the person know that the task is completed?	0	1	2	3

**Table 2:** Steps of the tea preparation activity with the outcomes and the help interpretation.

Activity Sequence Steps	Normal Behavior	Injected Errors	Help Injected	Help Interpretation
Step: Find the teabag. Expected task for this step: Memory retrieval. Possible organization errors: Omission and commission.	The subject requests and finds the teabag from the declarative memory.	The subject forgets and cannot retrieve the teabag.	Chunk activation to help the subject remember the teabag.	Verbal Help: One chuck activation boost.
		The subject confuses the teabag with the water. Similarity confusion injected between these two items.		Physical Help: More than one chunk activation.
		The subject confuses the teabag with something else.		Incapable for performing: More than two chunk activations.
Step: Let the tea rest for 3–4 min. Expected task for this step: Behavior execution and completion goal. Possible behavior errors: Completion error.	The subject executes this step successfully and moves to the next one.	The subject does a completion error and cannot complete this step. The utility probability to success and fail is injected.	Increase the utility success factor to help the subject complete the goal.	Verbal Help: Add extra utility value for successful firing of the production.
				Physical Help: If verbal help fails with the extra factor given, the physical help production runs with an extra utility value greater than the verbal help.
				Incapable of performing: The physical help failed.

**Table 3:** Comparison of the KTA and Serna et al.’s model with our framework values; mean values and standard deviations.

Level of AD	KTA Mean (SD)	Serna et al. Mean (SD)	Framework Mean (SD)
CDR 0.5—MCI	1.75 (2.21)	1.69 (1.12)	1.73 (1.32)
CDR 1—Mild AD	4.65 (3.73)	4.52 (1.72)	4.6 (2.27)
CDR 2—Moderate AD	9.81 (4.57)	9.87 (2.33)	9.67 (2.28)
CDR 3—Severe AD	13.88 (4.61)	13.84 (2.41)	13.36 (1.63)

## Data Availability

Not applicable.
